# An Urgent Call to Integrate the Health Sector into the *Post-2020 Global Biodiversity Framework*

**DOI:** 10.3390/ijerph20010861

**Published:** 2023-01-03

**Authors:** Simon King, Christopher J. Lemieux, Melissa Lem

**Affiliations:** 1 Park Doctor Pty Ltd., St. Lucia 3936, KwaZulu Natal, South Africa; 2 Department of Geography and Environmental Studies, Wilfrid Laurier University, Waterloo, ON N2L 3C5, Canada; 3 Department of Family Practice, University of British Columbia, Vancouver, BC V6T 1Z3, Canada

**Keywords:** biodiversity, human health, well-being, conservation, *Convention on Biological Diversity*, transformative change, mainstreaming

## Abstract

There is a rapidly closing window of opportunity to stop biodiversity loss and secure the resilience of all life on Earth. In December 2022, Parties to the United Nations (UN) *Convention on Biological Diversity* (CBD) will meet in Montreal, Canada, to finalize the language and terms of the *Post-2020 Global Biodiversity Framework* (*Post-2020 GBF*). The *Post-2020 GBF* aims to address the shortcomings of the previous *Strategic Plan on Biodiversity 2011–2020*, by introducing a Theory of Change, that states that biodiversity protection will only be successful if unprecedented, transformative changes are implemented effectively by Parties to the CBD. In this policy perspective, we explore the implications of the Theory of Change chosen to underpin the *Post-2020 GBF*, specifically that broad social transformation is an outcome that requires actors to be specified. We detail how the health sector is uniquely positioned to be an effective actor and ally in support of the implementation of the *Post-2020 GBF*. Specifically, we highlight how the core competencies and financial and human resources available in the health sector (including unique knowledge, skill sets, experiences, and established trust) provide a compelling, yet mostly untapped opportunity to help create and sustain the enabling conditions necessary to achieve the goals and targets of the framework. While by no means a panacea for the world’s biodiversity problems, we posit that explicitly omitting the health sector from the *Post-2020 GBF* substantially weakens the global, collective effort to catalyze the transformative changes required to safeguard biodiversity.

## 1. Introduction

The year 2023 marks the 30th anniversary of the multilateral United Nations (UN) *Convention on Biological Diversity* (CBD). Despite this major milestone, ecosystem degradation and biodiversity loss continue at unprecedented rates [1]. In a post-mortem reflection on the outcomes of the CBD’s previous decadal global strategic plan for biodiversity, *the Strategic Plan on Biodiversity 2011–2020* [2], the International Union for the Conservation of Nature (IUCN) concluded that “*despite an increase in policies and actions to support biodiversity, indicators show that the drivers of biodiversity loss have worsened and biodiversity further declined between 2011 and 2020. At the global level none of the 20 Aichi Biodiversity Targets agreed to by Parties to the CBD in 2010 have been fully achieved.*” (emphasis added by authors) [3]. The recent *Living Planet Report 2022* by the Word Wildlife Fund (WWF) further accentuated these conclusions, revealing that wildlife populations have declined by 69 % since 1970 and 1 million species are now threatened with extinction [4]. The global situation is considered so ominous that the Chair of the International Platform for Biodiversity and Ecosystem Services (IPBES), Sir Robert Watson, concluded that ongoing biodiversity loss is *“eroding the very foundations of our economies, livelihoods, food security, health and quality of life worldwide.”* [5]. Past policy failures, coupled with exacerbating geo-political challenges and climate change, means that there is a rapidly closing window of opportunity to stop biodiversity loss and secure the resilience of all life on Earth. 

Most people attribute better health to the healthcare system. However, research indicates that only 20% of human health is attributable to clinical care [1] and the remaining 80% depends on social, economic, and environmental determinants of health. The reality of the connection between human and environmental health is a lived experience today because of the recent pandemic. It has been a pillar of scientific medical practice since the beginning of the profession. It crosses all perspectives and cultures. Traditional indigenous ways of knowing, which include Natural or First Laws that view biospheric values as human values, also foreground the interconnectedness of human and ecosystem health [2]. The natural environment is a major determinant of the health of people and communities, but its influence is not always positive. Healthy environments improve health of humans, and indeed all life on Earth, whilst unhealthy or depleted environments can harm health. While definitions of “ecosystem health” vary, it has been suggested that healthy ecosystems exhibit states of ‘wholeness’ or ‘fullness of life’ (intactness), a maximum biological potential based on intact physical, chemical, and biological components and their interrelationships, such that it is resilient to withstand change and stressors see [3]. Biodiversity has been positioned as the single most important measure of natural system health [4,5], underpinning nature’s contributions to people [6,7], and integral to key development sectors that moderate health outcomes directly or indirectly, such as pharmacy, biochemistry, recreation and tourism, and the food system [7,8,9]. Indeed, it is estimated that more than half the world’s total Gross Domestic Product (GDP) is moderately or highly dependent on nature and its services [10]. As such, intact, natural ecosystems comprised of millions of species on land, freshwater, and in the ocean, have become a proxy for health, fundamental to the prosperity, sustainability, and quality of life of all people. 

Human society has the capacity to exert powerful anthropogenic forces. These forces can degrade and hasten biodiversity loss, damaging ecosystem health, which in health terms is known as a *pathogenic effect–that is, something that causes disease and harm*. The human capacity to degrade ecosystems is primarily why the CBD was enacted in 1993 [11]. With the support of 196 Parties (nations) today, the convention has near universal participation among the world’s countries and represents the best hope we have to address humanity’s pathogenic impact. Fortunately, humans also have the power to reduce biodiversity threats, stabilize, and foster recovery of biodiversity, thus promoting environmental health. In health terms, this is known as a *salutogenic effect–that is, factors that support or promote human health and well-being*. 

Stable, or healthy, ecosystems ultimately depend greatly on biodiversity. The relationship between biodiversity and human health is complex [12]. At a foundational level, biodiversity underpins the ecological functions and processes that give rise to the goods and services provided by ecosystems [13]. In addition to the provisioning (e.g., food, fresh water) and regulating (e.g., climate, pollination) services that ecosystems provide, an increasing body of evidence indicates that exposure to biodiverse environments, across the nature continuum from urban parks to remote parks and other forms of protected areas, can provide many health benefits, including improved mental health through reduced stress and anxiety [14,15,16] and physiological health through increased physical activity [17,18]. Biodiverse environments also provide important spaces that contribute to social well-being [19] and are critical to the development of children (e.g., better concentration, and experiences of environmental competence) [20].

It is now accepted that given current trajectories, the global goals for protecting biodiversity will only be achieved through far-reaching changes across economic, social, political, and technological factors [5,21,22]. This is recognized in the first draft of the CBD’s proposed *Post-2020 Global Biodiversity Framework* [23] (hereafter, the *Post-2020 GBF*), set to be agreed upon by the Parties to the CBD in Montreal, Canada, in December 2022. The *Post-2020 GBF* will guide global biodiversity conservation efforts over the next decade and beyond and specifically seeks to “*galvanize urgent and transformative action*” by all of society so that *“the trends that have exacerbated biodiversity loss will stabilize in the next 10 years (by 2030) and allow for the recovery of natural ecosystems in the following 20 years, with net improvements by 2050.”* [23]. 

*Ensuring the survival of large, biodiverse-rich natural ecosystems, is literally a public health initiative.* Biodiversity loss can have significant direct human health impacts if NCPs become impaired. The *Post-2020 GBF* is therefore a salutogenic social initiative–a self-proclaimed “ambitious” plan seeking to implement broad-based societal action. It also moves the biodiversity conservation community into new and difficult terrain. Specifically, the *Post-2020 GBF* recognizes the critical need for a *“whole-of-government and society approach”* and that *“…its implementation will be done in partnership among organizations at the global, national and local levels to leverage ways to build a momentum for success.*” [23]. While the first draft of the *Post-2020 GBF* communicates clear outcomes and goals and recognizes the need to involve a diversity of participants, it falls drastically short of identifying key participants that can and must be recruited to achieve these goals. The conservation community does not have the relevant capacity and expertise to manage a broad-based social initiative aimed at transforming the relationship human societies have with a determinant of health, but that capacity and those skills can be found, to a substantial extent, in the health community.

In this policy perspective, we propose that the health sector is a key, mission-critical partner to the biodiversity agenda. We propose that the persistent challenges that face biodiversity and the profound changes required to effectively address them demand that the health sector be recognized explicitly and intentionally as an important actor and ally in the effective implementation of the *Post-2020 GBF*. As a highly skilled, trusted, connected, and resourced partner, its potential to galvanize transformative change for biodiversity conservation is vast. We substantiate these claims on three interrelated lines of reasoning: 

First, the health sector is well-positioned to bring biodiversity into its sphere of influence and advance the Theory of Change (ToC) required to effectively achieve the goals and targets of the *Post-2020 GBF*. Explicitly identifying the health sector as an important actor and ally in the *Post-2020 GBF* will be necessary for its effective implementation. 

Second, the health sector can contribute core competencies and capabilities (including unique knowledge, skill sets, experiences, established trust and human and financial resources) to successfully enable the implementation of the *Post-2020 GBF*. These untapped resources present an opportunity to speed up, scale up, and sustain the biodiversity agenda. 

Third, deliberations leading up to the *Post-2020 GBF* clearly portray a desire and intent to overtly link health to biodiversity in the history of the biodiversity movement, yet this partnership remains absent in the text of the *Post-2020 GBF*. The time is now to mainstream partnerships across the human health and biodiversity sectors.

We revisit these three lines of reasoning below. Specifically, we link the CBD’s new ToC model to the health sector in theoretical and practical ways, including how strategically situating biodiversity as a human health resource may more adequately resource and compel society and decision-makers to effectively implement the *Post-2020 GBF*. We intersperse specific recommendations on where and how the health sector can be explicitly considered in the goals, milestones, and targets of the framework. Finally, we conclude by justifying the necessity of the health sector’s explicit and intentional recognition within the framework if Parties to the CBD truly aspire to effectively reverse ongoing biodiversity degradation and loss. While the issue of human health and biodiversity is vast and complex and cannot be addressed comprehensively in a policy perspective article such as this, our goal is to ignite this crucial and urgent conversation through the lines of reasoning presented below. 

## 2. Recommendation 1: Explicitly Identify the Health Sector as a Major Actor and Ally in the Effective Implementation of the *Post-2020 GBF*

To address deficiencies of previous strategic plans more effectively, the CBD has changed course and adopted a ToC approach in the development of the *Post-2020 GBF* [23]. At a fundamental level, the approach involves identifying beneficiaries and mapping out what needs to happen (actions) to support desired results, putting in place tools and solutions for mainstreaming, creating enabling conditions and adequate means of implementation (e.g., financial resources, human resources, technology) collectively within what is commonly referred to as a ‘ToC pathway’ [24,25,26]. 

ToC as a tool has great potential for use in the biodiversity conservation sector but is not yet widely used [25]. It is also not used with as much experience and sophistication as in other sectors. As Rice et al. noted recently, “*ToC use in conservation lags significantly behind many other sectors. In particular, the health, education and agriculture sectors possess rich empirical work evaluating the design and application of ToC, which offers several lessons for developing robust conservation ToC pathways.”* [25] Detailed evaluations of the use of ToC as a tool in the *Post-2020 GBF* have been published elsewhere in the literature (e.g., Rice et al.) and will not be reviewed here. What is relevant to this discussion is that the ToC model in the *Post-2020 GBF* is deficient in key details, especially with respect to the identification and role of key actors in its implementation (Table 1).

In evaluating the framework, we find that health is implicit in many assumptions, actions, and desired outcomes (including the ultimate end-goal of “Living in Harmony with Nature”). Yet, the first draft of the *Post-2020 GBF* falls acutely short of identifying the health sector as a key participant that must be recruited to achieve these goals (Table 2). Instead, in its current form, the framework takes a more ambiguous “catch-all” approach that refers to a variety of civic and private institutions, ranging from Indigenous peoples and local communities to youth groups. Of note, Sections H. and I. of the framework, which focus on implementation support mechanisms and enabling conditions, inexplicably omit the health sector from the wide range of conceivable partnerships required to support its implementation (Table 3).

As Rice et al. aptly noted, *“the greater the complexity of the problem, the greater an intervention’s need for multiactor engagement.”* With health as an ethos literally permeating the entire *Post-2020 GBF* (although indistinctly), the health sector, as a trusted and relevant messenger, is an indispensable ally for achieving its objectives. Surveys consistently position nurses and physicians as the world’s most trustworthy professionals [27]. If the *Post-2020 GBF* truly sets out to transform society’s relationship with ecosystem health then, simply put, it needs the help of the health sector. One potential avenue here is to adopt “nested” actor-based pathways [28], either now or in future derivatives work, to support implementation. While considered important to program monitoring and evaluation, nested actor-based pathways can also be used to increase capacity and participation, strengthen inter-departmental collaboration between government actors, facilitate the establishment of bridging organizations, identify “champions”, and strengthen the leadership required to drive initiatives [25]. Whether such actor-based pathways and specialist ToC models are included now or later, they must be linked to a provision in the overarching *Post-2020 GBF* (Table 4). If the health sector is not explicitly identified within the framework, these critical partnerships may be slower to form, or not form at all, thereby stalling efforts required to galvanize the resources necessary to support transformative action, some of which are detailed in Recommendation 2 below.

## 3. Recommendation 2: Leverage the Core Competencies and Resources of the Health Sector to Effectively Enable the Implementation of the *Post-2020 GBF*

As the *Post-2020 GBF* is setting out a plan to influence human society’s relationship with the construct of health, it needs to specifically identify the health sector as a key actor for future detailed pathways and ‘second-order’ ToCs to be developed for it. Given the TofC model adopted by the CBD, the aim of the *Post-2020 GBF* should be to channel every resource humanity can collectively muster in the most organized, coherent, and impactful way possible, to maximize our potential of realizing the vision of a human world “Living in Harmony with Nature.” Explicit omission of the health sector from the *Post-2020 GBF* substantially weakens its ability to do this. Following on Recommendation 1, we see two powerful leverage points where the health sector can unquestionably contribute to the *Post-2020 GBF:* (1) human resources; and, (2) financial resources. We discuss each of these critical leverage points below. 

*Leverage Point 1: Access to human resources.* Healthcare is one of the largest sectors worldwide. There are an estimated 12 million doctors in the world and more than 50 million other healthcare workers [29], or one healthcare professional for about every 125 people on Earth. In the U.S. in 2018, healthcare surpassed manufacturing and retail to become the largest source of employment [30]. This positions the health sector as a high-value domain that can be mined as appropriate for core competencies. Considering the repeated calls by the CBD to more effectively mainstream biodiversity across sectors, conservation will need to look for ways it can augment its own limited human resources and partner with others to do the work needed. 

Of the potential partners, medical doctors and nurses in particular have the training and capabilities to rapidly upskill and acquire new knowledge, strong communication skills, as well as trusted social positioning and status, to make meaningful contributions to the cause of biodiversity. Further, public health messaging already occurs in the health sector through formal initiatives and daily discussions that occur in millions of healthcare settings around the world and across the entire life span. These are excellent channels for distributing biodiversity messaging. Not only do health professionals have the competency and opportunity to help, but their work, professional identity, and ethical commitments, all make the health of the environment especially relevant to them, more so than any other professional sector in society. They are an obvious place to seek help (Table 5).

Doctors and nurses self-organize into professional associations, which have, for most of modern history, had their practices and activities controlled by regulators. Both these kinds of institutions have taken concrete steps, in policy and expectation, towards including environmental health in the professional scope and role of the health professions. For example, a position statement from the International Council of Nurses (ICN) states that as the global voice of nursing, ICN: *“Strongly believes that nurses have a shared responsibility to sustain and protect the natural environment from depletion, pollution, degradation and destruction.”* [31]. For its part, the German Medical Association includes the preservation of what amounts to biodiversity in their professional code of conduct of physicians: *“Physicians have a special responsibility to participate in the preservation of the natural foundations of life with regard to their importance for human health.”* [32]. Perhaps most prominently, the World Organization of Family Doctors (WONCA), a voluntary association of 118 member organizations in 131 countries, issued a declaration entitled *A Declaration ‘Calling for the Family Doctors of the World to Act on Planetary Health’* in 2019 with the goal to: *“bring awareness of planetary health to family doctors, highlight its relevance to their clinical practices, and motivate them to take action through a variety of channels.”* [33]. These statements and many others from associations of healthcare professionals with a special interest in the wellbeing of the natural systems on our planet, demonstrate that the human healthcare resource of the world is a legitimate candidate to recruit to the cause of biodiversity conservation.

The human resource potential within the health sector would be of interest even if it did not have special competencies of relevance. However, with its unique and relevant skill set, it represents a highly attractive opportunity. Indeed, those working to conserve biodiversity have a lot to learn from the health sector, especially the public health community who, for nearly 75 years, has been effectively advancing preventative healthcare through research, development of evidence-based policies, and administration of services including public educational programs. In fact, many challenges in integrating evidence into conservation decision-making have been documented [34,35], with numerous calls for the biodiversity conservation community to adopt health-based models for decision-making, including basing decisions on the best available evidence, and considering aspects of equity, efficacy, and effectiveness [36]. 

The salutogenic social movement and related ToC approach the *Post-2020 GBF* envisions will require a specific skill set to design, implement, and monitor and evaluate. These skill sets are rare in conservation but are abundant amongst the health profession. For example, medical sociologists, who study the relationship between social factors and health, are well positioned to inform strategies aimed at influencing the relationship between social factors (such as the built and natural environment, access to education, and social interactions) and biodiversity. Furthermore, participatory approaches (such as Participatory Action Research) and social engagement are nearly always integrated into health methods because this field was designed to serve the public interest [37,38]. These are skills and qualities that the biodiversity conservation community may dream of, yet are available to them. The challenge for the biodiversity conservation community is that its broken links with the health community make them difficult to reach, often operating out of sight of conservation professionals who remain unaware of their scope and potential.

Finally, today we are seeing the exciting potential of directly including biodiversity in day-to-day practice. Every day, millions of health practitioners provide advice and prescriptions to patients who are usually influenced by that advice to some degree. A “nature is medicine” movement has emerged in the U.S., Canada, and other countries to encourage doctors and other health care providers to write medical prescriptions to visit parks for physical and mental health benefits, gaining broad support within the medical community and receiving international recognition [39,40,41,42]. This movement represents a tangible, powerful example of how health professionals of the world can go about moving forward the societal transformation the *Post-2020 GBF* seeks to make, *in their day-to-day work.* The relevance here is in the sheer scale of untapped potential. All 12 million doctors across the globe may not prescribe nature, but even if one in ten does, the cumulative impact annually is measured in billions of people’s days in nature. No other sector is positioned to scale society-wide pro-environmental and health promotion initiatives so dramatically. This includes the biodiversity conservation community itself, who often fail to raise awareness on the threats of biodiversity loss, the benefits of safeguarding it, and promoting a sense of urgency for action. Of further interest to the biodiversity conservation community is the fact that leveraging health professionals for health-orientated messaging *actually works*. Indeed, research has shown that public support for policies improves when those policies are linked to both human and Planetary Health goals [43]. 

*Leverage Point 2: Access to financial resources.* Second, effective conservation will require a lot more money, and that money will need to come from somewhere. Successful implementation of the *Post-2020 GBF* will require governments and the private sector to scale up biodiversity finance and, at the same time, reduce finance flows that harm biodiversity. Biodiversity is pervasively and systematically undervalued or unvalued in policy and related decision-making. For example, while the Organization for Economic Co-operation and Development (OECD) estimates that ecosystem services delivered by biodiversity are worth USD 125–140 trillion annually [7] (more than 1.5 times the size of global GDP), investment in biodiversity conservation is universally considered inadequate [44,45,46]. Based on currently available data, global biodiversity finance is estimated at USD 78–91 billion per year (2015–2017 average) [47]. This is equivalent to only 0.1% of the global GDP. Furthermore, governments spend approximately USD 500 billion per year to support measures that are potentially harmful to biodiversity, an estimate that is considered conservative [47]. 

In line with the 2030 goals and 2050 milestones of the *Post-2020 GBF*, the United Nations Environment Program (UNEP) estimated in their 2021 *‘State of Finance for Nature Report’* that we need to triple investments in nature by 2030 and increase them fourfold between 2030 and 2050 to successfully tackle the interlinked climate and biodiversity crises [48]. This funding gap between what we currently spend and what we need to spend to halt biodiversity loss has been set at an average of USD 711 billion per annum [49]. 

Global healthcare spending, on the other hand, has risen steadily since 2000, and is now valued at USD 9 trillion annually, or around 10% of the global GDP [50]. To put this investment in context, the money required to safeguard the health of the environment, for the next decade under the *Post-2020 GBF*, is less money than we spend *every single year* on human health. This is a sobering statistic when we consider how much human health is determined by the environment. Put another way, if the health sector were to motivate for 7.5% more funding or find ways to save that 7.5% and redirect it to biodiversity conservation, the entire required financial resources for nature would be provided, forever. 

Saving 7.5% by improving health-seeking and health-service-delivering practices is undeniably achievable. A recent article by Shrank et al. revealed that 760 billion to 935 billion USD is wasted in the U.S. health care system alone, accounting for approximately 25 to 30% of total healthcare spending annually [51]. Even the most cautious analyses would place the annual waste in healthcare spending globally at USD $1.5 trillion. That number is more than ten times the annual spending required to protect and restore biodiversity, and 20 times the amount society currently spends on the health of the environment. This inequity is nothing short of staggering given the circumstances. 

Finally, it is worth noting that the COVID-19 pandemic showed how society can mobilize when our will is sufficient. The International Monetary Fund (IMF) reported that governments around the world have matched the Post-WWII debt load and have become more indebted than at any point in modern history (226 trillion USD) [52]. COVID-19 added 19.5 USD trillion to global debt [53]. Putting this in context, in response to a global public health threat, the world borrowed enough money to pay for the conservation of biodiversity for the rest of this century, in just two years. *Health-related objectives drive spending, even if the health sector is not directly doing the spending.*

Arguably the greatest risk in explicitly omitting the health sector as a named actor in the *Post-2020 GBF* (Recommendation 1) is that it will create barriers to cross-disciplinary partnerships of substantial impact (Recommendation 2). There are complex social, political, and economic barriers to address to ensure the redistribution of resources, but they are not without solutions when there is an underlying rationale linking such resources to Planetary Health. New, innovative research partnerships between conservation and health experts to support evidence-based medicine and practice would be required, including how to better consider the context, traditions, and ways of knowing of Indigenous communities. Integration of human health into national biodiversity strategies and action plans (NBSAPs) will also not be easy, and specific, measurable, achievable, relevant, and time-bound indicators currently available, or that could be feasible to develop (and to mobilize data for) in the near future, would be required for monitoring and evaluation. Potential outcomes indicators, including “Population benefiting from ecosystem services” and “Access to green/blue spaces” have been put forward by the CBD [54]. However, without the will and motivation of the health sector, it is doubtful that any of this work will be undertaken. If health professionals do not see biodiversity conservation as a health issue within their scope of concern (Table 6), it is unlikely they will work on addressing this resource inequity, and inadequate public resourcing for biodiversity conservation will persist. 

## 4. Conclusions

Biodiversity-related initiatives and interventions can have both positive and negative impacts on human health. Similarly, interactions between people and biodiversity can strongly influence population health and livelihoods, in both positive and negative ways, and the overall sustainability of health interventions [55]. Given this, since 2008, each biennial meeting of the Conference of the Parties (COP) to the CBD has called for and welcomed the strengthening of cooperation with the World Health Organization (WHO) on the nexus between biodiversity and human health [12,56,57] (see Appendix A). Yet, despite these repeated calls, as well as the recent *Kunming Declaration*, which was adopted by nearly 100 nations at CBD COP 15 (Part 1) in 2021 (and called on Parties to *“…mainstream the conservation and sustainable use of biodiversity in decision-making by recognizing its integral contribution for human wellbeing and health”* [58]), there remains no explicit goal or target related to human health in the *Post-2020 GBF*. 

If the ambitious aim of the *Post-2020 GBF* of driving forward a salutogenic social transformation in the relationship of human society to biodiversity is to be realized, then this needs to drastically speed up and scale up. Not only is the health sector influential, trusted, and well-resourced, but every human on the planet interacts with a healthcare provider as a ‘patient’ at some point. Health professionals have the training, thinking, and communication skills to persuade patients and decision-makers and frame their worldviews, starting at a young age. For biodiversity conservation professionals, influencing societal mindsets towards ‘pro-conservation’ states, or influencing ‘pro-environmental behaviour’, is new territory and poorly understood. To the world’s health professionals, however, this is business as usual.

Transformative change in support of halting ongoing biodiversity loss will no doubt require more than the specific recommendations described above. Our greatest concern, however, is that omitting an explicit and clear call to unify the health of people and Planetary Health will create a barrier that will unnecessarily undermine the ambitious societal transformation objective of the *Post-2020 GBF*. This may represent a lost opportunity and could also do harm at a time when urgent and transformative changes are most required. We take the strong position that mainstream recognition of the biodiversity-human health link and the sheer scale of influence that the health sector encompasses compels specific attention within the *Post-2020 GBF*. The relevance of the health sector to the goals of the *Post-2020 GBF*, and the financial and human resources available in the health realm, likely represent the largest untapped resource for conservation in securing the Earth’s biodiversity. Additionally, while recruiting the entire health system and convincing it to adopt the biodiversity problem within its ambit of concern is an incredibly ambitious task, the *Post-2020 GBF*’s ToC is itself ‘ambitious’. Biodiversity needs all the help that society can muster and there is no part of society more suited to working arm-in-arm with the biodiversity conservation community, than the health community.

## Figures and Tables

**Table 1:** Suggested revised health-inclusion statement and rationale for “Section D. Theory of Change” within the *Post-2020 GBF*.

Section of *Post-2020 GBF*: D. Theory of Change	
**Original wording in the *Post-2020 GBF*:** *“6. The framework’s theory of change assumes that transformative actions are taken…” [and] “7. The theory of change for the framework acknowledges the need for appropriate recognition of…”*	**Proposed new wording:** *“The framework’s theory of change acknowledges that biodiversity conservation is a public health initiative and highlights the need to make it relevant and attract the involvement of all professionals in the health sector.”*
**Rationale:** The ToC recognizes a need for substantial societal change but fails to identify specific actors that will enable and support the implementation of the *Post-2020 GBF*.	

**Table 2:** Suggested revised health-inclusion statement and rationale for “Section E. 2050 Vision and 2030 Mission” within the *Post-2020 GBF*.

Section of *Post-2020 GBF*: E. 2050 Vision and 2030 Mission	
**Original wording in the *Post-2020 GBF*:** *“9. The vision of the framework is a world of living in harmony with nature where: By 2050, biodiversity is valued, conserved, restored and wisely used, maintaining ecosystem services, sustaining a healthy planet and delivering benefits essential for all people.”*	**Proposed new wording:** *“9. The vision of the framework is a world of living in harmony with nature where: By 2050, biodiversity is valued, conserved, restored and wisely used, maintaining ecosystem services, and sustaining a healthy planet and people.”*
**Rationale:** The language links health to planet, but not to people. It refers to ecosystems delivering ‘benefits’ to people. We propose that this is a problematic message and tends to invite a utilitarian perspective where humanity focuses on what it can get from nature. We see a need to integrate and focus the message.	

**Table 3:** Suggested revised health-inclusion statement and rationale for “Section I. Enabling Conditions” within the *Post-2020 GBF*.

Section of *Post-2020 GBF*: I. Enabling Conditions	
**Original wording in the *Post-2020 GBF*:** *“15. It will require a participatory and inclusive whole-of-society approach that engages actors beyond national Governments, including subnational governments, cities and other local authorities (including through the Edinburgh Declaration), intergovernmental organizations, non-governmental organizations, indigenous peoples and local communities, women’s groups, youth groups, the business and finance community, the scientific community, academia, faith-based organizations, representatives of sectors related to or dependent on biodiversity, citizens at large, and other stakeholders.”*	**Proposed new wording:** *“15. It will require a participatory and inclusive whole-of-society approach that engages actors beyond national Governments, including subnational governments, cities and other local authorities (including through the Edinburgh Declaration), intergovernmental organizations and interdepartmental offices, non-governmental organizations, indigenous peoples and local communities, women’s groups, youth groups, the healthcare community, the business and finance community, the scientific community, academia, faith-based organizations, representatives of sectors related to or dependent on biodiversity, citizens at large, and other stakeholders.”*
**Rationale:** This section lists many potential actors to support the implementation of the framework, but nowhere is there an explicit mention of the health sector or health-related professions as actors.	

**Table 4:** Suggested new health-inclusion statement and rationale for “Section A. Background Information” within the *Post-2020 GBF*.

Section of *Post-2020 GBF*: A. Background Information	
**Original wording in the *Post-2020 GBF*:** *“Biodiversity and the benefits it provides, is fundamental to human wellbeing and a healthy planet.”*	**Proposed new wording:** *“Biodiversity is fundamental to the health and well-being of both people and planet.”*
**Rationale:** The original language in the *Post-2020 GBF* communicates a link between biodiversity and health in the frame-setting opening sentence. However, it divides biodiversity’s benefit, linking ‘healthy’ to ‘planet’, but ‘wellbeing’ to people. We recommend unifying this statement for clarity and strength.	

**Table 5:** Suggested new health-inclusion statements and rationale for “Section K. Outreach, awareness and uptake” within the *Post-2020 GBF*.

Section of *Post-2020 GBF*: K. Outreach, awareness and uptake
**Proposed new statements:** *“Successful execution of the framework will require a broad societal valuing of healthy ecosystem conditions and dynamics. Governments will need to invest in techniques to move society’s value system in this direction, including all levels of education. The health sector has a particularly relevant role to play in this regard.”* *“Biodiversity should be fully integrated into the training curricula for all health professionals at medical schools and training colleges, with a special focus on doctors and community care nursing training. Courses should be developed for post-graduate qualification for medical professionals to prepare them to prepare practitioners of all kinds with the competencies they will need to work more effectively on human and ecosystem health.”*
**Rationale:** The message in this section is echoed elsewhere, that ‘mainstreaming’ and generating awareness, participation and support is essential to effective implementation. The framework refers here to uptake by ‘all stakeholders’ but we propose that the health sector and its skill sets, and influence have a particular relevance to this sociological work and deserves mention.

**Table 6:** Suggested new health target and rationale for “Section G. 2030 Action Targets” within the *Post-2020 GBF*.

Section of *Post-2020 GBF*: G. 2030 Action Targets
**Proposed new target:** *“Target XX: Ensure that the health sector actively takes up biodiversity values and projects, through their official inclusion in curricula for health professionals, and creation of liaison roles between departments of health and the environment.”*
**Rationale:** Arguably, human health could be more explicitly recognized in several Targets. However, we focus our attention on targets focusing on “Tools and solutions for implementation and mainstreaming.” The message in this section of the targets is that health is a passive, down-stream factor impacted by the state of the environment, but not as a societal force that can be used to improve it. This message runs the risk of disenfranchising and alienating health professionals, communicating to them that they have no role to play. This is even more important because other actors (Indigenous Peoples, local communities, etc.) are identified as actors who can contribute to the goals. However, the health sector is not identified.

## Data Availability

Not applicable.

## Appendix A

**Table A1:** Notable milestones recognizing biodiversity and human health by the *Convention on Biological Diversity* (CBD) and/or the World Health Organization (WHO). All website links accessed on 12 November 2022.

Year	Milestone	Source
**1993**	CBD comes into force, noting *“that conservation and sustainable use of biological diversity is of critical importance for meeting the food, health and other needs of the growing world population, for which purpose access to and sharing of both genetic resources and technologies are essential.”*	CBD 1992 (http://www.un-documents.net/cbd.htm)
**2005**	First International Conference on Health and Biodiversity is held in Galway, Ireland.	
**2008**	COP nine called for the strengthening and cooperation with WHO and biodiversity and human health.	UNEP/CBD/ COP/9/INF/46 16 May 2008 (https://www.cbd.int/doc/meetings/cop/cop-09/information/cop-09-inf-46-en.pdf)
**2010**	20 Aichi Biodiversity Targets, part of the UN CBD *Strategic Plan for Biodiversity 2011-2020*, are released. Target 14 calls on Parties to the CBD to *“By 2020, ecosystems that provide essential services, including services related to water, and contribute to health, livelihoods and well-being, are restored and safeguarded, taking into account the needs of women, indigenous and local communities, and the poor and vulnerable.”*	CBD (https://www.cbd.int/aichi-targets/target/14)
**2012**	Parties at the eleventh meeting of the COP to the UN CBD requested the establishment of a joint work programme with WHO, in collaboration with relevant organizations and initiatives.	UNEP/CBD/COP/11/35 5 December 2012 (https://www.cbd.int/doc/meetings/cop/cop-11/official/cop-11-35-en.pdf)
**2014**	COP twelve adopts its first full decision on biodiversity and health and encourages Parties to *“consider the linkages between biodiversity and human health in the preparation of national biodiversity strategies and action plans, development plans, and national health strategies…”* and to *“…continue efforts under the joint work programme between the Secretariat and the World Health Organization…”.*	UNEP/CBD/COP/DEC/XII/21 17 October 2014 (https://www.cbd.int/doc/decisions/cop-12/cop-12-dec-21-en.pdf)
**2014**	WHO adopts resolution WHA67.12 and releases the *Health in All Policies* (HiAP) report. HiAP is defined as *“an approach to public policies across sectors that systematically takes into account the health implications of decisions, seeks synergies and avoids harmful health impacts in order to improve populations health and health equity”.* The HiAP approach recognizes that *“Many of the determinants of health and health inequities in populations have social, environmental, and economic origins that extend beyond the direct influence of the health sector and health policies. Thus, public policies in all sectors and at different levels of governance can have a significant impact on population health and healthequity.”*	Resolution WHA67.12 (2014) (https://apps.who.int/iris/bitstream/handle/10665/162850/A67_R12-en.pdf)
**2015**	WHO and the Secretariat of the *Convention on Biological Diversity* signed a Memorandum of Understanding (MOU) to strengthen collaboration and, with relevant partners, capitalize on their respective scientific and technical expertise on the links between health and biodiversity (co-chaired by both Parties).	CBD and WHO 2015 (https://www.cbd.int/doc/agreements/agmt-who-2015-07-23-mou-en.pdf)
**2015**	WHO and the CBD release the report entitled *Connecting global priorities: biodiversity and human health: a state of knowledge review.* The review details how biodiversity and ecosystems provide essential services upon which humans depend and warn of the need to stem the tide of infections and non-communicable diseases arising from the destruction of nature.	WHO and CBD 2015 (https://www.who.int/publications/i/item/9789241508537)
**2016**	COP thirteen adopts its second full decision on biodiversity and human health considering the implications of the findings of the joint publication on state of knowledge review on biodiversity and human health. The decision invites governments and other parties to promote the *“understanding of health-biodiversity linkages with a view to maximizing health benefits, addressing trade-offs, and where possible, addressing common drivers for health risks and biodiversity loss”* and *“decides to consider biodiversity and human health interlinkages when addressing the follow-up to the Strategic Plan for Biodiversity 2011–2020 and the Aichi Biodiversity Targets.”*	CBD/COP/ DEC/XIII/6 14 December 2016 (https://www.cbd.int/decisions/cop/13/6)
**2018**	At the 71st World Health Assembly, WHO releases the report entitled, *Health, environment and climate change: human health and biodiversity* by the Director General.	29 March 2018 A71/11 (https://www.who.int/publications/i/item/A71_11)
**2021**	The WHO, through its Department of Environment, Climate Change and Health (ECH), the International Union for the Conservation of Nature (IUCN), and the Friends of Ecosystem-based Adaptation (FEBA) network are establishing a new expert working group (EWG) on Biodiversity, Climate, One Health and Nature-based Solutions.	IUCN (https://www.iucn.org/news/ecosystem-management/202103/new-who-iucn-expert-working-group-biodiversity-climate-one-health-and-nature-based-solutions)
**2021**	The First Draft of the *Post-2020 Global Biodiversity Framework* is released. The 2050 Vision and 2030 Mission of the framework notes that *“The vision of the framework is a world of living in harmony with nature where: By 2050, biodiversity is valued, conserved, restored and wisely used, maintaining ecosystem services, sustaining a healthy planet and delivering benefits essential for all people.” * Nature’s contributions to human health are recognized, often in a non-explicit manor, in several targets, including **Target 5** (harvesting), **Target 11** (air and water quality, hazards), and **Target 15** (biotechnology). **Target 12** recognizes human health the most explicitly, noting: “Increase the area of, access to, and benefits from green and blue spaces, for human health and well-being in urban areas and other densely populated areas.”	CBD/WG2020/ 3/3 5 July 2021 (https://www.cbd.int/doc/c/abb5/591f/2e46096d3f0330b08ce87a45/wg2020-03-03-en.pdf)
**2021**	Part 1 of the CBD COP 15 *Kunming Declaration* to keep the political momentum of the negotiations delayed by the COVID-19 pandemic. The Declaration calls upon the parties to “mainstream” the conservation and sustainable use of biodiversity in decision-making by recognizing its integral contribution for human well-being and health.	The Kunming Declaration (https://www.cbd.int/doc/c/df35/4b94/5e86e1ee09bc8c7d4b35aaf0/kunmingdeclaration-en.pdf)
**2022**	CBD COP 15 Part 2 to be held in Montreal, Canada in December. It is expected that the Post-2020 *Global Biodiversity Framework* will be agreed to by COP at this event.	CBD/COP 15 (https://www.cbd.int/meetings/COP-15)
